# Rates and Risk Factors for On‐Treatment Mortality Among a Cohort of Adults Treated for Drug‐Sensitive Tuberculosis: Analysis of Data From the Adherence Support Coalition to End Tuberculosis Consortium in Five Countries

**DOI:** 10.1111/tmi.70043

**Published:** 2025-10-03

**Authors:** Amare W. Tadesse, Noriah Maraba, Jason Alacapa, Katya Gamazina, Tanyaradzwa Dube, Baraka Onjare, Norma Madden, Salome Charalambous, Christopher Finn McQuaid, Degu Jerene, Katherine L. Fielding

**Affiliations:** ^1^ Department of Infectious Disease Epidemiology and International Health, London School of Hygiene & Tropical Medicine (LSHTM) London UK; ^2^ The Aurum Institute Johannesburg South Africa; ^3^ KNCV Tuberculosis Plus Manila Philippines; ^4^ Program for Appropriate Technology in Health (PATH) Kyiv Ukraine; ^5^ KNCV Tuberculosis Plus Dar es Salaam Tanzania; ^6^ KNCV Tuberculosis Plus The Hague the Netherlands; ^7^ School of Public Health University of Witwatersrand Johannesburg South Africa

**Keywords:** adults, cohort analysis, drug‐sensitive tuberculosis, mortality, risk factors

## Abstract

**Background:**

Tuberculosis remains a leading cause of death globally, particularly in countries with high tuberculosis and HIV burdens. Disruptions caused by the COVID‐19 pandemic may have further impacted tuberculosis outcomes. This study examines on‐treatment mortality and associated risk factors in five countries.

**Method:**

We conducted a secondary analysis of data from ASCENT cluster‐randomised trials of digital adherence tools for improved adherence involving 23,799 adults with drug‐sensitive tuberculosis in South Africa, Tanzania, Ethiopia, the Philippines, and Ukraine. Analyses were conducted separately by country. Mortality rates were measured from treatment initiation to the earliest of 6 months, death, or loss to follow‐up. Cox regression models (with random effects or robust standard errors for clustering) assessed the associations between mortality and HIV status, ART use, tuberculosis diagnosis type, and calendar periods (COVID‐19 pandemic and conflict in Ukraine).

**Results:**

Mortality rates ranged from 7.6 (Ethiopia) to 23.2 (Tanzania) and 23.3 (Ukraine) per 100 person‐years. Higher mortality was associated with: older age in all countries (age < 30 versus ≥ 60 years, adjusted rate ratio [aRR] ranging from 2.38 to 6.57 by country); HIV status (positive versus negative, aRR ranging from 1.44 to 2.98 by country); tuberculosis diagnosis type (clinical vs. bacteriological, aRR 1.5–1.6 in Ethiopia, Tanzania and South Africa); extrapulmonary tuberculosis (aRR 1.44 to 1.60 in Ukraine and Tanzania). ART versus HIV‐positive not on ART was linked to lower mortality in South Africa and Ukraine but not in Tanzania. Analyses suggested possible mortality variations by calendar period.

**Conclusion:**

Our findings suggest variability in tuberculosis mortality across settings, influenced by HIV/ART and diagnosis type. The high mortality rates across countries may reflect underlying causes or potential misdiagnoses. Further investigation into these factors may be needed to improve tuberculosis outcomes globally.

## 1 Introduction

Mortality due to tuberculosis (TB) remains a challenge globally, with an estimated 1.09 million HIV‐negative and 161,000 people with HIV dying from TB in 2023. The global End TB strategy goal is to reduce the annual number of deaths by 75% against the 2015 baseline. However, only an 8.7% reduction has been achieved from 2015 to 2022 [1]. The presence of the COVID‐19 pandemic in 2020–2022 resulted in the reduction of TB testing and access to TB services globally [2]. Modelling studies have also predicted that the effect of the disruption in TB services during the pandemic will lead to an increase in TB deaths even in the years after the pandemic has ended [3, 4].

HIV is a well‐documented major risk factor for TB‐associated mortality in countries with high HIV prevalence [5]. However, with wide availability, ART has led to a 44%–77% reduction in mortality among people living with HIV [6]. According to a systematic review, risk factors for TB mortality differ according to HIV prevalence. In high HIV prevalence settings, in addition to HIV positivity, reported risk factors were smear‐negative disease and malnutrition. In low HIV prevalence settings, risk factors include sputum smear‐positive disease, alcohol, and substance misuse [7].

The Adherence Support Coalition to End TB (ASCENT) consortium conducted five pragmatic cluster randomised trials to evaluate the effect of digital adherence technologies with differentiated response on TB treatment outcomes in South Africa, Tanzania, Ethiopia, the Philippines, and Ukraine [8, 9]. These trials involved large cohorts of adults being treated in routine settings for TB, with baseline data collection and follow‐up, making this one of the few studies to report such detailed cohort data across diverse, high‐burden contexts. South Africa, Tanzania, Ethiopia, and the Philippines are listed among the 30 high TB‐burden countries globally, while Ukraine is listed among the 30 high‐burden multidrug/rifampicin‐resistant countries [10]. Understanding risk factors of mortality enables country TB prevention and care programmes to plan appropriate interventions, targeting groups at greatest risk of death and subsequently reducing mortality in their settings.

In this study, we aim to report on‐treatment mortality and assess whether HIV/ART status, clinical TB diagnosis, and extrapulmonary TB increase mortality, based on secondary analyses of the ASCENT trials, among adults with drug‐sensitive TB in South Africa, Tanzania, Ethiopia, the Philippines, and Ukraine. Additionally, we explored whether mortality differed by calendar period due to the COVID‐19 pandemic and the war in Ukraine.

## 2 Methods

### 2.1 Design and Population

We conducted a secondary analysis using data from the five ASCENT trials. We included adult persons (aged 18 years or above) on treatment for drug‐sensitive TB (DS‐TB), who had complete treatment outcome data from the TB register. We excluded participants with no (missing) documented outcomes, not evaluated, or who had changed treatment for drug‐resistant tuberculosis (DR‐TB).

### 2.2 Setting

The ASCENT trial results are reported elsewhere. In brief, the trials, conducted in Ethiopia, the Philippines, South Africa, Tanzania and Ukraine, were cluster‐randomised at the health facility level (rayon in Ukraine) and evaluated digital adherence interventions to improve treatment outcomes. There was no impact of the intervention on improving treatment outcomes in all five countries [11, 12]. These countries, among the top 30 countries with a high burden of TB, were chosen to reflect different epidemiological, socioeconomic, geographical, infrastructural and health system factors [8, 9]. The health facilities included both small and large, rural and urban facilities.

### 2.3 Data Source and Variables

All data for this study came from the ASCENT research database. We used routine treatment information recorded on standard paper‐based TB registers (in Ethiopia, South Africa and Tanzania) and captured in the research database by the research team. Data from the electronic TB register in the Philippines and Ukraine were imported into the research database. Baseline (treatment start) data included age, sex, HIV and ART status at baseline, TB diagnosis (clinical or bacteriologically confirmed), calendar period of enrolment into the trial, type of facility (hospital versus non‐hospital/health centre in Tanzania) and geographical area. Data on HIV and ART status were not shared in the electronic download from the Philippines. In the Ethiopian study, additional data on marital status, education, housing structure and household assets were collected through participant interviews.

### 2.4 Definition of Study Outcome

The study outcome, for all countries except for the Philippines, was time to death on TB treatment, measured from the start date of treatment. Follow‐up time was censored at the date of treatment outcome or the start date plus 180 days, whichever came first. In the Philippines, the date of treatment outcome may not always reflect when the event occurred, but rather when the outcome was reported, and so the analysis was based on on‐treatment mortality risk.

### 2.5 Statistical Analyses

Separate analyses were conducted by trial, using Stata version 18.

For participant data from Ethiopia, we used polychoric regression to calculate a dual‐based wealth index based on household assets, toilet facilities, type of cooking fuel, water source and people sleeping/room to describe individuals' relative socioeconomic position (SEP), grouped into quintiles [13].

The overall and covariate‐specific TB mortality rates per 100 person‐years of follow‐up (PY) were calculated. We grouped baseline covariates based on their proximity to the outcome. Distal exposures were age (grouped) and sex, for all countries, and in Ethiopia, SEP, marital status, and education. The intermediate exposure was HIV/ART status and proximal exposures were TB diagnosis (bacteriological vs. clinical), type of TB (pulmonary TB [PTB] or extra‐pulmonary [EPTB]), and previous TB. Covariate adjustment was based on variables distal to the particular exposure. We used Cox regression for time to death and logistic regression for mortality risk, with a random effect for health facility/rayon. When there were convergence problems with the random effects Cox model, robust standard errors were used to adjust for clustering for the calendar period analyses, and the Poisson model with random effects was used for the analysis of baseline covariates.

For all countries, except the Philippines, we further assessed if mortality varied by calendar period, with a particular interest in the effect of COVID‐19 and the war in Ukraine on mortality, both of which coincided with periods of follow‐up. We used a lexis expansion for calendar period and time from treatment start as the underlying time frame (due to mortality changing rapidly with this time frame), adjusting for age, sex, HIV/ART status, clinical versus bacteriological diagnosis, and in addition SEP, marital status, and education in Ethiopia.

### 2.6 Ethical Considerations

Informed consent was sought from all trial participants in Ethiopia, while a waiver of consent was granted for the remaining four trials, as no patient identifiers were collected, and there was no direct patient contact occurred with the research team. The trials were approved by: the London School of Hygiene and Tropical Medicine Ethics Committee (19135 and 19120), United Kingdom; WHO Ethical Review Committee (ERC.0003296 and ERC.0003297), Switzerland; the Addis Ababa City Administration Health Bureau Public Emergency and Health Research Directorate Institutional Review Board (A/A/H/B/1/978/227), and Oromia Regional Health Bureau Public Emergency and Health Research Directorate Institutional Review Board (BEFO/HBTFH/1–16/10322), Ethiopia; the Philippines (Single Joint Research Ethics Board SJREB 2019–57); South Africa (University of Witwatersrand Human Research Ethics Committee 200102); Tanzania (Tanzania Medical Research Coordinating Committee (MRCC) at National Institute for Medical Research, Dar es Salaam NIMR/HQ/R.8a/Vol.IX/3431); and Ukraine MOH of Ukraine (Ukraine IRB 2019–33), PATH Research Ethics Committee (1522814–2). Data were analysed anonymously and kept confidential following the ethical principles of the Helsinki Declaration.

## 3 Results

A total of 23,799 adult participants with TB across the five trials were started on TB treatment over the period from 1 December 2020 to 5 August 2022; follow‐up ended on 25 January 2023. Overall, 36% (8537/23,799) were female, and 21% (5090/23,799) were under 30 years of age, with a younger population in Ethiopia. The proportion of participants with a history of previous TB ranged from 3% in Tanzania to 22% in Ukraine. Bacteriologically confirmed TB was reported in approximately 55% of participants (13,009/23,799). The proportion of HIV‐positive participants varied by country, from 13% in Ethiopia to 62% in South Africa, and ART coverage was above 90% in Ethiopia, South Africa, and Ukraine, and 74% in Tanzania. The majority of participants had PTB. Educational attainment and marital status data were available in Ethiopia only, with the commonest levels being less than primary education (37%) and married or cohabiting (47%) (Table 1).

**TABLE 1:** Baseline characteristics of study participants with drug‐sensitive TB, by country (*n* = 23,799).

Variable	Categories	Ethiopia *n* (%)	Tanzania *n* (%)	South Africa *n* (%)	The Philippines *n* (%)	Ukraine *n* (%)
Total	n	3827	6442	4103	6459	2968
Sex	Male	2269 (59.3)	4055 (63.0)	2571 (62.7)	4354 (67.4)	2013 (67.8)
	Female	1558 (40.7)	2387 (37.0)	1532 (37.3)	2105 (32.6)	955 (32.2)
Age, years	Median (IQR)	30 (24–40)	45 (33, 59)	41 (33, 50)	47 (33, 58)	44 (37, 54)
	18–29	1886 (49.3)	1061 (16.5)	643 (15.7)	1257 (19.5)	263 (8.9)
	30–39	836 (21.8)	1402 (21.8)	1263 (30.8)	1131 (17.5)	752 (25.3)
	40–49	504 (13.2)	1416 (21.9)	1117 (27.2)	1293 (20.0)	921 (31.0)
	50–59	273 (7.1)	987 (15.3)	637 (15.5)	1338 (20.7)	575 (19.4)
	≥ 60	328 (8.6)	1576 (24.5)	443 (10.8)	1440 (22.3)	457 (15.4)
Previous TB	No/unknown Yes	3571 (93.3) 256 (6.87)	6254 (97.1) 188 (2.9)	3626 (88.4) 477 (11.6)	5636 (87.3) 823 (12.7)	2322 (78.2) 646 (21.8)
Disease site	PTB EPTB	3827 (100) 0 (0)	5497 (85.3) 945 (14.7)	3607 (87.9) 496 (12.1)	6459 (100) 0 (0)	2351 (79.2) 617 (20.8)
HIV/ART status	Negative Positive (no/unk ART) Positive (on ART)	3337 (87.4) 15 (0.4) 468 (12.2)	4900 (76.1) 394 (6.1) 1148 (17.8)	1542 (37.6) 221 (5.4) 2340 (57.0)	NA	2308 (77.8) 31 (1.0) 629 (21.2)
ART coverage^a^	On ART	468/483 (96.9)	1148/1542 (74.4)	2340/2561 (91.4)		629/660 (95.3)
TB Diagnosis	Bacteriological	2480 (64.8)	2821 (43.8)	2413 (58.8)	3727 (57.7)	1568 (52.8)
	Clinical	1347 (35.2)	3621 (56.2)	1690 (41.2)	2732 (42.3)	1400 (47.2)
Education (highest level)	None Less than primary Primary Secondary or higher	528 (13.9) 1394 (36.7) 695 (18.3) 1183 (31.1)	NA	NA	NA	NA
Marital status	Single Single living alone Married/cohabiting Separated/widowed	965 (25.4) 634 (16.7) 1783 (46.9) 418 (11.0)	NA	NA	NA	NA

*Note*: Missing data: Ethiopia: HIV/ART *n* = 7; education *n* = 27; marital status *n* = 27.

Abbreviations: ART, antiretroviral therapy; EPTB, extra‐pulmonary TB; NA not applicable; PTB, pulmonary TB; unk, unknown.

^a^ ART coverage was calculated as the proportion of people with TB/HIV co‐infection who were receiving antiretroviral therapy (ART) at the time of starting TB treatment.

Among the 23,799 participants, 6% (1,525/23,799) died during treatment. The mortality rate varied across countries: 7.6 (130/1,718) in Ethiopia, 23.2 (620/2,668) in Tanzania, 15.8 (292/1,846) in South Africa, and 23.3 (293/1,256) in Ukraine, per 100 PY. Notably, the mortality rates/risks increased with older age and were higher among people living with HIV, clinically diagnosed TB (except Ukraine), and for those with EPTB (Table 2, Tables S1 and S2a). In Ethiopia, in unadjusted analyses, lower levels of education and being separated or widowed were associated with higher mortality rates. In the distal models, older age was associated with increased mortality rate/risk (*p* < 0.001, in all countries). In Ethiopia, once adjusting for age and sex, the adjusted rate ratios (aRR) for marital status and education were attenuated to 1 (Table S2b). We therefore decided to present the adjusted effect of SEP, as an overall measure of relative wealth, in the distal model, which showed no relationship with mortality.

**TABLE 2:** On‐treatment mortality rates 100 person‐years among categories of adults with drug‐sensitive TB, by country.

		Ethiopia		Tanzania		South Africa		The Philippines		Ukraine	
		Deaths/pyrs	Rate per 100 pyrs	Deaths/pyrs	Rate per 100 pyrs	Deaths/pyrs	Rate per 100 pyrs	Deaths/N	Risk	Deaths/pyrs	Rate per 100 pyrs
Overall		130/1718	7.6	620/2668	23.2	292/1846	15.8	190/6459	2.9%	293/1256	23.3
Sex	Male Female	83/1017 47/702	8.2 6.7	386/1678 234/991	230 23.6	189/1146 103/700	16.5 14.7	135/4354 55/2105	3.1% 2.6%	209/846 84/410	24.7 20.5
Age, years	18–29	29/858	3.4	71/449	15.8	26/292	8.9	21/1257	1.7%	10/115	8.7
	30–39	26/374	6.9	103/587	17.5	59/577	10.2	17/1131	1.5%	65/315	20.6
	40–49	27/224	12.0	115/601	19.1	76/505	15.0	35/1293	2.7%	74/396	18.7
	50–59	16/120	13.3	93/409	22.8	62/283	21.9	35/1338	2.6%	69/245	28.2
	≥ 60	32/142	22.6	238/622	38.3	69/188	36.7	82/1440	5.7%	75/186	40.4
HIV status	Negative	101/1503	6.7	414/2051	20.2	98/697	13.9	NA		181/998	18.1
	Positive (no ART)	0/6	0.0	52/157	33.1	22/94	23.4			26/2.5	1046
	Positive (on ART)	29/206	14.1	154/460	33.5	173/1054	16.4			86/256	33.6
TB Diagnosis	Bacteriological	66/1117	5.9	191/1203	15.9	133/1096	12.1	104/3727	2.8%	161/655	24.6
	Clinical	64/602	10.6	429/1466	29.3	159/750	21.2	86/2732	3.1%	132/601	22.0
Previous TB	No/unknown Yes	122/1602 8/116	7.6 6.9	604/2592 16/76	23.3 21.0	254/1637 38/21	15.5 18.2	150/5636 40/823	2.7% 4.9%	236/988 57/268	23.9 21.1
Disease site	PTB EPTB	NA (all PTB)		471/2303 149/365	20.4 40.8	248/1620 44/225	15.3 19.5	NA (all PTB)		200/1003 93/253	19.9 36.8
Care facility	Not hospital Hospital	NA		174/1066 446/1602	16.3 27.8	NA		NA		NA	
Geographic area	Rural Urban	4/116 126/1602	3.4 7.9	236/1220 384/1449	19.3 26.5	69/347 223/1499	19.9 14.9	65/2527 125/3932	2.6% 3.2%	95/348^a^ 198/907	27.2 21.8

*Note*: Missing data: Ethiopia: HIV/ART *n* = 7; education *n* = 27; marital status *n* = 27; socio‐economic position *n* = 114. See Table S1 for mortality rates by socio‐economic position, highest education level and marital status.

Abbreviations: ART, antiretroviral therapy; EPTB, extra‐pulmonary TB; NA, not applicable; PTB, pulmonary TB; Pyrs, person‐years.

^a^ semi‐rural.

In adjusted analyses, HIV co‐infection was associated with increased mortality in Ethiopia (aRR: 1.60, 95% confidence interval (CI): 1.00–2.57), Tanzania (aRR: 1.79, 95% CI: 1.49–2.14), South Africa (aRR: 1.44, 95% CI: 1.11–1.86), and Ukraine (aRR: 2.98, 95% CI: 2.30–3.85). Compared to being HIV‐negative, those HIV‐positive on ART had a lower adjusted RR than those HIV‐positive not on ART in South Africa and Ukraine, whereas the adjusted RRs were similar in Tanzania (Table 3).

**TABLE 3:** Adjusted analyses of individual‐level risk factors for on‐treatment mortality, among adults with drug‐sensitive TB, by country.

			Ethiopia^d^ (*n* = 3713)	Tanzania (*n* = 6442)			South Africa (*n* = 4103)		The Philippines (*n* = 6459)		Ukraine (*n* = 2968)	
			RR (95% CI)	*p*	RR (95% CI)	*p*	RR (95% CI)	*p*	OR (95% CI)	*p*	RR (95% CI)	*p*
**Distal factors (model 1)**												
Age grouped (years)		18–29	1	< 0.001	1	< 0.001	1	< 0.001	1	< 0.001	1	< 0.001
		30–39	2.10 (1.23–3.57)		1.14 (0.84–1.55)		1.15 (0.73–1.84)		0.91 (0.48–1.74)		2.39 (1.23–4.65)	
		40–49	3.68 (2.17–6.25)		1.28 (0.95–1.73)		0.68 (1.07–2.62)		1.64 (0.94–2.88)		2.20 (1.13–4.26)	
		50–59	3.99 (2.16–7.36)		1.46 (1.07–2.00)		2.32 (1.46–3.68)		1.65 (0.95–2.87)		3.20 (1.64–6.21)	
		≥ 60	6.57 (3.92–11.0)		2.38 (1.82–3.12)		3.72 (2.36–5.87)		3.54 (2.16–5.81)		4.48 (2.31–8.67)	
Sex		Male Female	1 0.97 (0.67–1.39)	0.85	1 1.03 (0.87–1.21)	0.76	1 0.95 (0.75–1.21)	0.70	1 0.79 (0.57–1.10)	0.16	1 0.82 (0.63–1.06)	0.12
SEP		Poorest	1	0.38								
		Second	1.43 (0.82–2.43)									
		Middle	0.81 (0.44–1.47)									
		Fourth	1.05 (0.61–1.83)									
		Wealthiest	0.99 (0.58–1.71)									
Effect of HIV and HIV/ART status^a^ (models 2a, 2b)												
2a	HIV status	Negative Positive	1 1.60 (1.00–2.57)	0.041	1 1.79 (1.49–2.14)	< 0.001	1 1.44 (1.11–1.86)	0.005	NA		1 2.98 (2.30–3.85)	< 0.001
2b	HIV/ART status	Negative Positive‐not on ART Positive‐ART	NA‐ no deaths in the pos‐no ART group		1 1.79 (1.32–2.41) 1.79 (1.47–2.18)	< 0.001	1 2.06 (1.27–3.33) 1.39 (1.07–1.80)	0.005	NA		1 37 (24–58) 2.27 (1.72–2.99)	< 0.001
**TB diagnosis/type (models 3a, 3b)**												
3a	TB diagnosis type^b^	Bacteriological Clinical	1 1.46 (1.03–2.08)	0.036	1 1.56 (1.30–1.88)	< 0.001	1 1.64 (2.28–2.09)	< 0.001	1 0.97 (0.71–1.32)	0.83	1 0.94 (0.73–1.20)	0.61
3b	TB type^c^	PTB EPTB	All PTB		1 1.60 (1.31–1.95)	< 0.001	1 1.30 (0.93–1.80)	0.13	All PTB		1 1.44 (1.08–1.92)	0.014

Abbreviations: ART, antiretroviral therapy; CI, confidence interval; EPTB, extra‐pulmonary TB; NA, not applicable; OR, odds ratio; PTB, pulmonary TB; RR, rate ratio; SEP, socio‐economic position (relative measure).

^a^ Adjusted for age grouped and sex (and SEP for Ethiopia).

^b^ Adjusted for age grouped, sex, and HIV (and SEP for Ethiopia), except for the Philippines, which is adjusted for age grouped and sex only.

^c^ Adjusted for age grouped, sex, and HIV (pos/neg).

^d^ Ethiopia: All results are from the Poisson model with random effects, due to the non‐convergence of the Cox model with random effects.

Clinical diagnosis of TB was associated with higher mortality in Ethiopia (aRR: 1.46, 95% CI: 1.03–2.08), Tanzania (aRR: 1.56, 95% CI: 1.30–1.88), and South Africa (aRR: 1.64, 95% CI: 2.28–2.09). Extrapulmonary TB was associated with an increased mortality rate in Tanzania (aRR: 1.60, 95% CI: 1.31–1.95) and Ukraine (aRR: 1.44, 95% CI: 1.08–1.92) (Table 3). The analysis of calendar periods revealed some fluctuations in mortality rates over time, though no strong statistical evidence of differences (Table 4).

**TABLE 4:** Mortality analyses for calendar period, by country.

Current calendar period—lexis expansion	Ethiopia		Tanzania		South Africa		Ukraine	
	Deaths/pmths	Rate per 1000 pmths	Deaths/pmths	Rate per 1000 pmths	Deaths/pmths	Rate per 1000 pmths	Deaths/pmths	Rate per 1000 pmths
–09/21	23/215	10.7	59/131	44.9	29/137	21.2	36/124	29.1
10/21–12/21	31/416	7.5	130/579	24.0	61/387	15.7	82/360	22.8
01/22–03/22	36/470	7.7	179/794	22.5	76/586	13.0	77/408	18.9
04/22–06/22	23/470	4.9	143/776	18.4	89/618	6.2	64/328	19.5
07/22–9/22	14/365	3.8	83/666	12.5	37/486^a^	4.9	34/288^a^	11.8
10/22—	3/126	2.4	17/255	6.6				
	aRR (95% CI)	*p*	aRR (95% CI)	*p*	aRR (95% CI)	*p*	aRR (95% CI)	*p*
–09/21	1.60 (0.90–2.85)	0.12	0.93 (0.67–1.26)	0.15	1.01 (0.65–1.57)	0.54	0.68 (0.45–1.04)	0.05
10/21–12/21	1.44 (0.84–1.49)		0.99 (0.78–1.25)		0.94 (0.67–1.31)		1.14 (0.72–1.59)	
01/22–03/22	1.50 (0.93–2.44)		1.24 (0.99–1.55)		0.83 (0.61–1.14)		1.13 (0.81–1.58)	
04/22–06/22	1		1		1		1	
07/22–9/22	0.87 (0.43–1.76)		1.06 (0.80–1.39)		0.75 (0.50–1.10)^a^		1.35 (0.87–2.11)^a^	
10/22—	1.05 (0.33–3.35)		1.55 (0.89–2.71)		—		—	

*Note*: Ethiopia: adjusted for time since starting treatment as underlying time frame and fixed effects for age, sex, HIV (pos/neg) and disease classification, SEP —used Cox model with robust standard errors. Tanzania, South Africa, Ukraine: adjusted for time since starting treatment as underlying time frame and fixed effects for age, sex, HIV (pos/neg) and disease classification, —used Cox model with random effects. Ukraine—war started in February 2022.

Abbreviations: aRR, adjusted rate ratio; CI, confidence interval; pmths, person‐months.

^a^ For South Africa and Ukraine the latest calendar period is ≥ 07/22.

## 4 Discussion

The study revealed large differences in TB‐related mortality across five countries, with Tanzania and Ukraine exhibiting the highest mortality rates of 23.2 and 23.3 deaths per 100 person‐years, respectively. Expectedly, HIV‐positive individuals not on ART had the highest mortality rate, compared with HIV‐negative individuals, and higher mortality was observed among older age groups, particularly those aged 60 years and above. In addition, having a clinical TB diagnosis versus bacteriologically confirmed TB and extra‐pulmonary TB versus pulmonary TB were associated with higher mortality rates. Although TB mortality fluctuated over time, neither COVID‐19 nor the war in Ukraine fully explained these variations, with no strong evidence for COVID‐19 and weak evidence for the war's impact.

These findings further confirm that having HIV co‐infection is the greatest risk factor across the countries and this is worsened when patients do not receive ART. This highlights a need for additional measures to mitigate the impact of HIV on TB treatment outcomes. The higher mortality rate among persons with clinically diagnosed and extra‐pulmonary TB suggests a need for further investigation into underlying reasons. One possible explanation is that delays or inaccuracies in TB diagnosis could be higher in these groups, possibly leading to death due to undiagnosed serious health conditions such as lung malignancies or cardiovascular diseases. Further exploration of immediate causes of death through mortality audits or post‐mortem examinations can yield additional insights into the causes of death among such patients. The higher mortality rate among older patients can also be due to unrecognised underlying conditions such as diabetes, hypertension, or other cardiovascular risk factors.

Variable mortality rates across the five countries align with findings from systematic reviews, which indicate that mortality during and after tuberculosis treatment varies by region, with high‐burden countries experiencing higher death rates [7, 14, 15]. These findings are consistent with global evidence, showing variability in TB outcomes influenced by differing healthcare systems, HIV co‐infection rates, and socioeconomic factors. A previous systematic review has documented that TB mortality is region‐specific, with high‐burden areas displaying mortality rates similar to what was observed in our study, particularly in settings with limited resources and higher HIV prevalence [7].

Unsurprisingly, older age was associated with increased on‐treatment mortality, with individuals over 60 years facing the highest risk across all countries studied. This finding is supported by a study conducted in Tanzania and Ethiopia, which found that older people with TB patients had a significantly increased risk of death [15, 16]. Similarly, a systematic review of risk factors for TB mortality in South Africa [15] underscores that older age groups consistently experience higher TB‐related mortality across different settings, a trend seen in other high‐burden countries [7]. This aligns with findings in previous studies where older patients in high‐burden TB settings showed increased mortality due to the presence of comorbidities, such as HIV and diabetes, which further complicate treatment outcomes [17, 18], as well as challenges in early diagnosis and treatment [7]. These findings highlight the critical need for TB programmes to focus on active case finding among elderly populations, given their disproportionately high mortality rates. Tailored approaches are essential, including comprehensive screening to identify multi‐morbidities that may remain undiagnosed and providing more intensive monitoring and treatment support for these vulnerable groups. Moreover, integrating TB care with broader health services to address comorbidities and ensuring the timely initiation of treatment could play a significant role in reducing mortality. Strengthening the capacity of healthcare workers to manage complex cases and updating programme guidelines to account for the unique challenges older TB patients face are necessary steps to improve outcomes in this high‐risk population.

We found HIV status to be an important risk factor for mortality. This is consistent with findings from multiple studies across the globe, particularly in regions where HIV prevalence is high [6, 7, 14, 19]. People with TB/HIV are at a much higher risk of mortality due to their compromised immune systems [6, 14, 16]. Our study also found that compared to being HIV‐negative, those on ART in South Africa and Ukraine had lower RR than those not on ART, though this was not the case in Tanzania, where ART coverage was also lower. This highlights the critical need for timely ART initiation to improve outcomes. While our findings confirm that HIV co‐infection is an important risk factor for TB mortality, the high mortality observed among HIV‐negative individuals also demands attention. This may partly reflect that HIV‐negative individuals were older compared to those living with HIV in all countries except Ethiopia, and coupled with comorbidities such as diabetes or cardiovascular disease, likely contributes to their increased mortality. Addressing these challenges requires better management of comorbidities and targeted interventions to tackle broader determinants of health, ensuring equitable TB outcomes across populations.

Studies from sub‐Saharan Africa, including Ethiopia, suggest that individuals with TB who have lower socio‐economic status may face greater barriers to accessing timely and effective treatment, increasing their risk of mortality [14, 15, 20]. However, our findings from Ethiopia showed no relationship between our measure of relative socio‐economic position and mortality. While the universal access to TB services in Ethiopia may mitigate some of the disparities linked to the socio‐economic status of the population, the high mortality rates we documented could still be indirectly influenced by the overall low socio‐economic status of the population, which may exacerbate challenges such as poor nutrition, housing conditions, education, and comorbidities. These findings suggest that, in the Ethiopian context, whilst relative socio‐economic position may not differentiate outcomes, the overall low socio‐economic conditions of the cohort likely contribute to adverse TB outcomes, alongside clinical and healthcare‐related factors. Further research is warranted to explore these dynamics and inform targeted interventions to address the broader determinants of health.

Clinical diagnosis of TB was common in all five studies. Our findings revealed that people with clinically diagnosed TB had higher mortality compared to those with bacteriologically confirmed TB, after adjustment for HIV and other factors. This could be due to a longer time to diagnosis, resulting in more advanced or complicated disease presentations at the time of diagnosis. Recent studies suggested that delayed or less accurate clinical diagnosis can lead to inappropriate or delayed treatment initiation, which significantly worsens prognosis [14, 15]. Misdiagnosis or delays in clinical confirmation highlight a need for improved diagnostic infrastructure, particularly in settings where clinical diagnosis remains common.

We found higher mortality rates among people with EPTB compared to those with PTB in Tanzania and Ukraine. This observation is consistent with global trends, where EPTB, especially forms such as TB Meningitis, is known for diagnostic challenges and delays in treatment initiation, leading to worse outcomes [7, 15, 19]. EPTB's atypical presentation often contributes to diagnostic delays, as seen in studies from South Africa and Ethiopia, where delayed or missed diagnoses significantly impact survival [7, 14, 19]. The higher mortality in EPTB cases in our study highlights the need for improved diagnostic tools and earlier detection. EPTB often requires more complex and prolonged treatment regimens [21], and in resource‐limited settings, this complexity can lead to suboptimal management, further increasing mortality risk.

We observed small fluctuations in TB mortality by calendar period, after adjustment for time since treatment start. In the absence of this adjustment, the earlier calendar periods were more likely to be in their first 2 months of TB treatment, a critical window where mortality risk is greatest. We hypothesised that the COVID‐19 pandemic may result in increased mortality by calendar period. The pandemic disrupted healthcare systems globally, diverting resources away from TB programmes and delaying diagnosis and treatment [3, 4]. The adjusted model for Ethiopia showed that the TB mortality rate was relatively higher during peak waves of COVID‐19 (January 2022), indicating a possible negative impact on TB care services, though statistical evidence was weak. Recent studies in South Africa and Ethiopia have shown that delayed care during the pandemic worsened TB outcomes, contributing to increased mortality during certain periods [22, 23]. Third, some deaths may have been directly attributed to COVID‐19, especially in countries like Ukraine, where healthcare systems were overwhelmed by the dual burden of TB and COVID‐19. Additionally, the ongoing conflict in Ukraine, which began in February 2022, may have further reduced access to care, compounding the challenges in managing TB and driving higher mortality. These factors collectively underscore the importance of resilient healthcare systems, particularly in high‐burden TB settings, to mitigate the impact of external shocks like pandemics or conflict on TB care continuity.

Our study has important strengths and limitations. A key strength is the inclusion of large, diverse cohorts from five countries with different tuberculosis epidemiological profiles, allowing us to explore mortality patterns across various settings. The use of a causal framework to guide our analyses adds rigour to the findings and helps provide a deeper understanding of the factors driving TB mortality. Furthermore, tracking mortality across calendar periods enabled us to capture changes in TB outcomes over time, offering valuable insights into temporal trends. However, the study has limitations. The reliance on routine data meant we had limited covariates, except for Ethiopia, which may have constrained our ability to adjust for confounding factors. Additionally, we were unable to conduct a time‐to‐death analysis for the Philippines, and the absence of HIV data for this cohort limits our ability to assess the impact of co‐infections on mortality. Our study cohort consisted of outpatients eligible for the adherence intervention, which likely has led to an underestimation of overall mortality. This is because in‐patients, who were excluded from our analysis, likely faced higher mortality rates due to the greater severity of their illness. Despite these limitations, our findings are highly relevant to real‐world conditions, as they reflect the outcomes of patients receiving treatment within routine outpatient care settings, rather than under the controlled conditions of clinical trials. These insights provide important evidence on TB mortality across diverse contexts, supporting global efforts to improve TB outcomes.

This study highlights significant variability in TB‐related mortality across diverse settings, identifying key risk factors such as HIV co‐infection (particularly among individuals not receiving ART), older age, clinical TB diagnosis, and EPTB, all of which are associated with increased mortality. These findings underscore the urgent need for timely ART initiation, better integration of TB and HIV services, enhanced early diagnostic tools and infrastructure, tailored treatment support for elderly populations, and comprehensive screening for comorbidities in resource‐limited settings. The COVID‐19 pandemic may have further exacerbated TB mortality, emphasising the importance of building resilient health systems capable of maintaining essential TB services during crises and future pandemics. While this study provides valuable insights into TB mortality, further research is needed to better understand better immediate causes of death, including socio‐economic drivers of mortality, improve diagnostic tools, and assess the impact of global disruptions such as pandemics and conflict on TB outcomes. Addressing these gaps is critical to reducing TB mortality and advancing toward global TB elimination targets.

## Conflicts of Interest

The authors declare no conflicts of interest.

## Supporting information


**Data S1:** tmi70043‐sup‐0001‐Supinfo.docx.
