# Supplementary material for: Rates and Risk Factors for On‐Treatment Mortality Among a Cohort of Adults Treated for Drug‐Sensitive Tuberculosis: Analysis of Data From the Adherence Support Coalition to End Tuberculosis Consortium in Five Countries
Source: Trop Med Int Health. 2025 Oct 3;30(12):1363–73. doi: 10.1111/tmi.70043 (PMC12675320; doi:10.1111/tmi.70043)
Supplement: Supplementary file 1 — Data S1: tmi70043‐sup‐0001‐Supinfo.docx. [file TMI-30-1363-s001.docx]

**Supplement for: Tadesse et al Rates and risk factors for on-treatment mortality among a cohort of adults treated for drug-sensitive tuberculosis: analysis of data from the Adherence Support Coalition to End Tuberculosis consortium in five countries**

Contents

[Results 2](#_Toc190005020)

[Table S1 Mortality rates for socio-economic position, education and marital status in Ethiopia 2](#_Toc190005021)

[Table S2a Univariable analyses of individual-level risk factors for mortality, by country 3](#_Toc190005022)

[Table S2b : Adjusted analyses of individual-level risk factors for mortality, by country, adjusted for age and sex 5](#_Toc190005023)

# Results

## Table S1 Mortality rates for socio-economic position, education and marital status in Ethiopia

| **Variable** | **Coding** | **Deaths/pyrs** | **Rate per 100 pyrs** |
| --- | --- | --- | --- |
| SEP | Poorest  Second  Middle  Fourth  Wealthiest | 25/336  31/331  19/334  26/334  27/331 | 7.4  9.4  5.7  7.8  8.1 |
| Education (highest level) | None  Less than primary  Primary  Secondary or higher | 33/233  45/625  18/315  34/533 | 14.2  7.2  5.7  6.4 |
| Marital status | Single  Single living alone  Married/cohabiting  Separate/widowed | 18/438  20/284  71/799  21/185 | 4.1  7.1  8.9  11.4 |

Pyrs person-years; SEP socio-economic position

## Table S2a Univariable analyses of individual-level risk factors for mortality, by country

|  |  | **Ethiopia^1^** | **Tanzania** | **South Africa** | **The Philippines** | **Ukraine** |
| --- | --- | --- | --- | --- | --- | --- |
| **N** |  | **3827** | **6442** | **4103** | **6459** | **2968** |
|  |  | **RR (95% CI)** | **RR (95% CI)** | **RR (95% CI)** | **OR (95% CI)** | **RR (95% CI)** |
| Sex | Male  Female | 1  0.82 (0.57-1.17) | 1  1.00 (0.85-1.19) | 1  0.88 (0.69-1.12) | 1  0.82 (0.60-1.12) | 1  0.85 (0.66-1.10) |
| Age, years | <30  30-39  40-49  50-59  ≥60 | 1.0  2.06 (1.21-3.49)  3.56 (2.21-6.02)  3.93 (2.13-7.23)  6.69 (4.04-11.0) | 1.0  1.14 (0.84-1.55)  1.28 (0.95-1.73)  1.46 (1.07-2.00)  2.38 (1.90-3.11) | 1.0  1.16 (0.73-1.84)  1.69 (1.08-2.64)  2.34 (1.47-3.70)  3.75 (2.38-5.91) | 1.0  0.90 (0.47-1.72)  1.65 (0.94-2.88)  1.65 (0.95-2.86)  3.51 (2.14-5.76) | 1.0  2.40 (1.23-4.7)  2.25 (1.16-4.36)  3.26 (1.68-6.33)  4.42 (2.28-8.56) |
| HIV status | negative/unknown  Positive | 1  2.04 (1.35-3.08) | 1  1.57 (1.32-1.86) | 1  1.20 (0.94-1.53) | NA | 1  2.40 (1.89-3.07) |
| HIV ART status | negative/unknown  Positive not ART  Positive on ART | No events: HIV/ no ART group | 1  1.53 (1.14-2.06)  1.58 (1.30-1.91) | 1  1.76 (1.09-2.82)  1.15 (0.90-1.48) | NA | 1  31 (20-47)  1.84 (1.42-2.40) |
| TB diagnosis | Bacteriological  Clinical | 1  1.80 (1.28-2.54) | 1  1.79 (1.49-2.14) | 1  1.77 (1.39-2.25) | 1  1.19 (0.89-1.60) | 1  1.04 (0.81-1.32) |
| Type of TB | PTB  EPTB | NA: all PTB | 1  1.70 (1.39-2.07) | 1  1.28 (0.92-1.78) | NA: all PTB | 1  1.96 (1.53-2.52) |
| Past TB | No/unknown  Yes | Does not converge | 1  0.85 (0.52-1.41) | 1  1.11 (0.79-1.57) | 1  1.72 (1.21-2.46) | 1  0.84 (0.63-1.12) |
| Education | None  Less than primary  Primary  Secondary or higher | 1  0.51 (0.32-0.80)  0.40 (0.23-0.71)  0.45 (0.28-0.73) | NA | NA | NA | NA |
| Marital status | Single  Single living alone  Married/cohabiting  Separate/widowed | 1  1.72 (0.91-3.24)  2.16 (1.29-3.63)  2.77 (1.47-5.19) | NA | NA | NA | NA |
| SEP | Poorest  Second  Middle  Fourth  Wealthiest | 1  1.26 (0.74-2.13)  0.76 (0.42-1.39)  1.04 (0.60-1.81)  1.09 (0.64-1.89) | NA | NA | NA | NA |

Missing data: Ethiopia: HIV/ART n=7 ; education n=27; marital status n=27; socio-economic position n=114

^1^ Analyses based on Poisson model with random effects due to non-convergence of the Cox model with random effects

RR rate ratio, OR odds ratio, CI confidence interval, SEP socio-economic position (relative measure), PTB pulmonary TB, EPTB extra-pulmonary TB, ART antiretroviral therapy

When adjusting for age and sex the RRs for education are attenuated to 1: 0.75 (0.47-1.19), 0.68 (0.37-1.25) and 0.78 (0.47-1.33) for less than primary, primary and secondary or higher, respectively vs none.

When adjusting for age and sex the RRs for marital status are attenuated to 1: 1.54 (0.81-2.92), 1.32 (0.76-2.27) and 1.28 (0.65-2.53) for single living alone, married/cohabiting, separate/widowed, respectively vs single (not living alone)

## Table S2b : Adjusted analyses of individual-level risk factors for mortality, by country, adjusted for age and sex

|  |  | **Ethiopia^1^** | **Tanzania** | **South Africa** | **The Philippines** | **Ukraine** |
| --- | --- | --- | --- | --- | --- | --- |
| **N** |  | **3827** | **6442** | **4103** | **6459** | **2968** |
|  |  | **RR (95% CI)** | **RR (95% CI)** | **RR (95% CI)** | **OR (95% CI)** | **RR (95% CI)** |
| HIV status | negative/unknown  Positive | 1  1.57 (1.02-2.43) | 1  1.79 (1.49-2.4) | 1  1.44 (1.11-1.86) | NA | 1  2.98 (2.30-3.85) |
| HIV ART status | negative/unknown  Positive not ART  Positive on ART | No events: HIV/ no ART group | 1  1.79 (1.32-2.41)  1.79 (1.47-2.18) | 1  2.06 (1.27-3.33)  1.39 (1.07-1.80) | NA | 1  37 (24-58)  2.27 (1.72-2.99) |
| TB diagnosis | Bacteriological  Clinical | 1  1.47 (1.04-2.09) | 1  1.62 (1.35-1.95) | 1  1.69 (1.33-2.15) | 1  0.97 (0.71-1.32) | 1  1.05 (0.82-1.33) |
| Type of TB | PTB  EPTB | NA: all PTB | 1  1.60 (1.31-1.96) | 1  1.34 (0.96-1.86) | NA: all PTB | 1  2.11 (1.64-2.73) |
| Past TB | No/unknown  Yes | 1  0.75 (0.37-1.53) | 1  0.78 (0.48-1.30) | 1  1.06 (0.75-1.50) | 1  1.44 (0.99-2.11) | 1  0.81 (0.61-1.09) |
| Education | None  Less than primary  Primary  Secondary or higher | 1  0.75 (0.47-1.19)  0.68 (0.37-1.25)  0.79 (0.47-1.33) | NA | NA | NA | NA |
| Marital status | Single  Single living alone  Married/cohabiting  Separate/widowed | 1  1.54 (0.81-2.92)  1.32 (0.76-2.27)  1.28 (0.65-2.53) | NA | NA | NA | NA |
| SEP | Poorest  Second  Middle  Fourth  Wealthiest | 1  1.43 (0.84-2.43)  0.81 (0.44-1.47)  1.05 (0.61-1.83)  0.99 (0.58-1.71) | NA | NA | NA | NA |

^1^ education n=3800; marital status n=3800; SEP n=3713. Analyses based on Poisson model with random effects due to non-convergence of Cox model with random effects
